# High-Purity Copper Recovery from Copper Sludge via Oxidative Refining Using a FeO–CaO–SiO_2_ Slag System

**DOI:** 10.3390/ma18174137

**Published:** 2025-09-03

**Authors:** Eunmi Park, Minji Kim, Young-Min Kim, Yong Hwan Kim

**Affiliations:** 1 Incheon Technopark, Incheon 21999, Republic of Korea; 2 Korea Institute of Industrial Technology, Incheon 21999, Republic of Koreaymkim77@kitech.re.kr (Y.-M.K.)

**Keywords:** recycling, copper sludge, oxidative refining, FeO–CaO–SiO_2_ slag, pyrometallurgy, impurity removal

## Abstract

The rapid increase in electronic waste poses a significant environmental issue, with copper-rich residues considered among the most valuable fractions. Extracting copper of high purity from these materials is critical for advancing sustainable resource utilization. In this work, an oxidative refining approach employing a FeO–CaO–SiO_2_ slag matrix was investigated to purify copper-bearing sludge. The method facilitated stable slag generation and ensured distinct separation between the metallic phase and slag. Although Fe and Si were removed effectively at relatively low processing temperatures, complete removal of Sn and S occurred only at 1300 °C, with traces of copper oxides persisting in the refined alloy. Overall, the findings suggest that the proposed slag system offers a reliable strategy for producing high-purity copper from secondary sources, underscoring its relevance in sustainable recycling of copper-enriched wastes.

## 1. Introduction

Copper (Cu) plays an essential role in the electrical and electronic industries because of its excellent conductivity, good thermal resistance, and favorable mechanical properties. According to projections by the International Energy Agency (IEA), worldwide copper demand is expected to rise from 25.9 million tonnes in 2023 to 32.6 million tonnes in 2035, representing an increase of about 26% [1]. Because copper resources are limited and mining activities cause significant environmental burdens, recycling has emerged as a crucial approach with both economic and ecological benefits. Nevertheless, efficient copper recovery remains challenging. A major difficulty arises from the need to separate copper from complicated mixtures containing base metals (e.g., Fe, Sn, Ni, Zn) together with precious metals (e.g., Pd, Au, Ag) [2,3,4], as well as non-metallic components like glass, plastics, and ceramics. If residual impurities remain, they can seriously compromise the quality of recovered copper and restrict its use in advanced industrial applications. Hence, refining technologies that can selectively eliminate both metallic and non-metallic contaminants are essential to obtain high-purity copper that satisfies the strict requirements for reuse in electronic and electrical manufacturing [5,6,7].

The refining stage is a vital component in the pyrometallurgical recycling process, aiming to purify and concentrate the recovered metals to ensure their quality and market value. In this stage, impurities and contaminants are selectively eliminated, while valuable metals are refined to achieve high purity levels [5,8,9]. The oxidative refining process operates according to the principles illustrated in the Ellingham diagram, which shows the formation of metal oxides as a function of partial pressure of oxygen (pO_2_) and temperature [10]. Base metals have a greater tendency to oxidize compared to valuable metals, making them more prone to oxidation and removal during refining. This stage is critical to obtain metals that satisfy industrial standards and can be reused within manufacturing processes. Among refining methods, oxidative refining has emerged as an effective approach for enhancing purity and concentrating metals in pyrometallurgical recycling [11]. The process selectively oxidizes impurities such as Fe, aluminum (Al), Si, and lead (Pb), while preserving and enhancing the concentration of valuable metals [12].

Slag refers to a molten oxide mixture composed of oxides such as ferrous oxide (FeO) and silica (SiO_2_), classified into acidic, basic, and neutral groups. Acidic oxides, such as SiO_2_ and alumina (Al_2_O_3_), form long polyions when melted, leading to high viscosities and low solubilities for other acidic oxides, posing challenges in copper refining. Adding basic oxides like calcia (CaO) and magnesia (MgO) to acidic slags breaks down these polyions, reducing viscosity and increasing solubility for acidic oxides. Basic slags also have lower melting points. Neutral oxides like FeO and Cu_2_O lower the melting point and viscosity of slag, enhancing the refining process’s efficiency and quality. Ferrous silicate melts, widely used in primary copper smelting and converting, face limitations such as the need to control magnetite formation and a narrow molten composition range. Their acidic nature makes impurity removal difficult. The Mitsubishi converter introduced calcium ferrite (CaO-based) slags, which prevent magnetite formation and form a molten Cu_2_O-CaO-Fe_3_O_4_ slag, improving the process [13]. Calcium ferrite slags have lower viscosity and specific gravity, better impurity removal, and reduced copper losses. Despite these advantages, calcium ferrite slags have drawbacks, particularly in smelting. They have low SiO_2_ solubility, complicating the smelting of high-silica concentrates, and their aggressive behavior towards ‘mag-chrome’ (MgO–Cr_2_O_3_) refractories leads to frequent repairs and higher costs. To address these issues, extensive research over the past decade has focused on developing a more suitable slag type. Ferrous calcium silicate (FCS) slags, also known as olivine ((Fe,Ca)_2_SiO_4_) slags, have emerged as a potential alternative. FCS slags offer a compromise between the properties of calcium ferrite and fayalite slags, making them a possible replacement for fayalite slags in copper smelting [14].

Besides pyrometallurgical routes, hydrometallurgical and electrorefining methods have also been applied to recover copper from secondary resources. Hydrometallurgy, based on acid leaching with solvent extraction and electrowinning, operates at low temperatures but consumes large amounts of reagents and generates liquid waste, limiting large-scale use [15,16,17,18]. Electrorefining yields high-purity copper and is established in primary production [19,20,21,22], yet it requires high energy and high-quality anodes, making it less suitable for low-grade feeds. In contrast, pyrometallurgical refining can process heterogeneous inputs, operates more quickly, and effectively removes impurities at high temperatures.

This study investigates the effectiveness of FeO–CaO–SiO_2_ slag systems in the oxidative refining of crude metal recovered from copper-containing sludge. The primary objective is to enhance impurity removal and achieve efficient phase separation to improve the purity of the recovered Cu alloy. The 40FeO–30CaO–30SiO_2_ slag systems were selected to be investigated for their low melting point and viscosity, which are critical for slag formability and metal separation.

## 2. Materials and Methods

Copper-containing sludge was processed through high-temperature smelting, which liquefied the metallic phases and enabled their separation from slag. During this step, organic residues such as plastics and resins were thermally decomposed into gaseous by-products. At the same time, non-metallic components and minor elements—including calcium (Ca), phosphorus (P), silicon (Si), and aluminum (Al)—partitioned into the slag phase.

**Figure 1:**
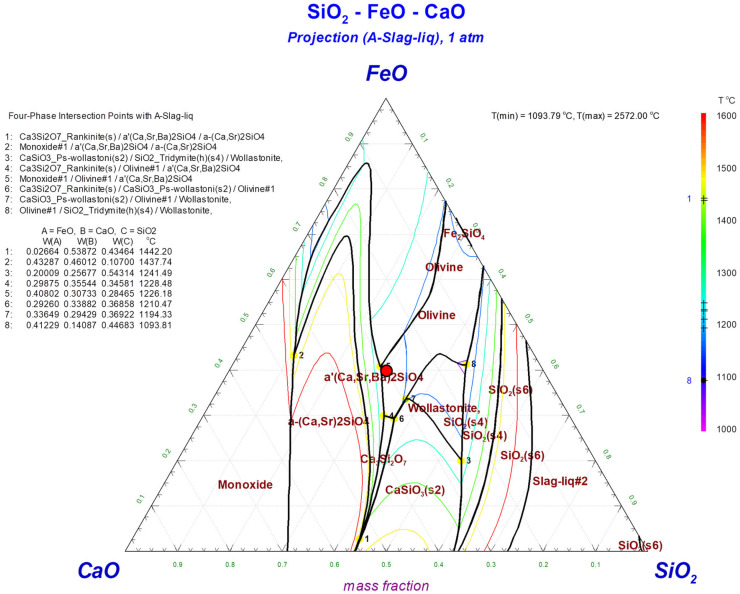
Phase diagram of FeO–CaO–SiO_2_.

Following smelting, the separated Cu-based metals were subjected to an oxidative refining process to remove residual impurities. A 150 g Cu-based master alloy obtained from the smelting process was prepared and introduced into a high-density alumina crucible with a size of 47Φ × 120 mm. Slag formers, including CaO and SiO_2_, were added during the process to form the 40FeO–30CaO–30SiO_2_ slag system (marked in Figure 1). The 40FeO–30CaO–30SiO_2_ slag composition was chosen based on the FeO–CaO–SiO_2_ phase diagram (Figure 1). This composition provides a relatively low melting temperature and moderate viscosity compared with other regions of the system, which are favorable for stable slag formation and smooth separation from the metallic phase. For the refining process, a gas mixture of oxygen (O_2_) and nitrogen (N_2_) was used. The master alloy was heated using a MoSi_2_ heater system with a heating/cooling rate of 5 °C/min. When the temperature reached the desired processing temperature, the mixture of O_2_ and N_2_ gases was directly injected into the melt through the alumina tube with a 1:1 ratio, with a total gas flow rate of 2 L/min for 10 min. The process temperature was varied between 1200 °C and 1300 °C to investigate its effect on the oxidative refining process.

The microstructure of the refined metals was analyzed using scanning electron microscopy (SEM, Quanta 200 FEG, FEI Company, Hillsboro, OR, USA). Phase formation in the refined metals was characterized by X-ray diffraction (XRD, SmartLab 9 kW, Rigaku, Tokyo, Japan). The impurity content of the refined metals was assessed through inductively coupled plasma (ICP, Integra XL DUAL, GBC Scientific Equipment Pty., Ltd., Keysborough, Australia) spectroscopy to quantify iron (Fe), tin (Sn), and silicon (Si), while sulfur (S) was measured using a carbon/sulfur (C/S) analyzer (CS-800, Eltra GMBH, Haan, Germany). For ICP analysis, approximately 1 g of alloy sample was digested using standard acid dissolution procedures (aqua regia or HF) to ensure complete dissolution of all components. The solutions were filtered, diluted to a final volume of 100 mL with deionized water, and further diluted prior to ICP-OES measurement.

## 3. Results and Discussions

**Figure 2:**
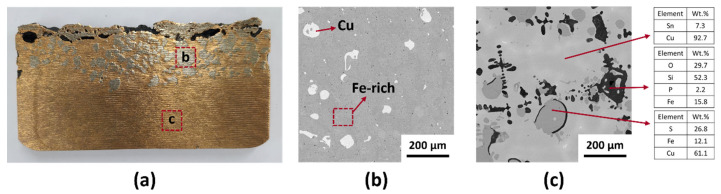
Crude metal recovered from copper-containing sludge by the arc smelting process. (**a**) Macrograph and (**b**,**c**) SEM micrograph of the crude metal.

The crude metal was recovered from copper-containing sludge using the arc smelting process. As shown in Figure 2a, the crude metal consists of distinct upper and lower phases. To better understand the composition of each phase, SEM analysis was conducted. Figure 2b presents the microstructure of the upper part of the sample, which shows an Fe-rich matrix with dispersed Cu droplets. These droplets result from liquid-phase separation, where Cu and Fe remain unmixed during cooling. In contrast, Figure 2c displays the microstructure of the lower part of the sample, which is primarily composed of a Cu–rich phase containing approximately 7 wt.% Sn. The high solubility of Sn in Cu at elevated temperatures, as indicated by the Cu–Sn phase diagram, explains the significant Sn content. Within the Cu–rich matrix, phases composed of Cu, Fe, and S were observed, alongside dendritic structures containing Si, Fe, P, and O. The behavior of Cu–Fe alloys is influenced by their positive mixing enthalpy and the presence of metastable miscibility gaps in the phase diagram, which lead to liquid-phase separation during solidification. Consequently, the Cu phase remains distinct from the Fe matrix. Thus, it demonstrates that the crude metal consists of Fe–rich and Cu–rich phases. Image analysis revealed that the crude alloy obtained after smelting consisted of distinct Fe–rich and Cu–rich phases, occupying 15.5% and 84.5% of the total volume, respectively (Figure 2a). The chemical composition of each phase was analyzed by XRF, and their mass fractions were calculated based on phase proportions. From these data, the overall chemical composition of the crude alloy was estimated, as summarized in Table 1. The alloy was mainly composed of 71.5 wt.% Cu and 16.9 wt.% Fe, with minor amounts of Sn (8.7 wt.%), P (1.3 wt.%), Si (0.7 wt.%), and S (1.0 wt.%). These values provide an approximate baseline composition for evaluating the refining behavior in subsequent oxidative refining experiments. Due to the heterogeneous microstructure of the crude alloy, the obtained composition should be regarded as an estimated bulk value. Due to the incomplete separation of the two phases, achieving a clean separation of Cu and Fe is challenging. Thus, further oxidative refining is necessary to promote the oxidation of impurities, ultimately enhancing the purity of the Cu alloy.

**Figure 3:**
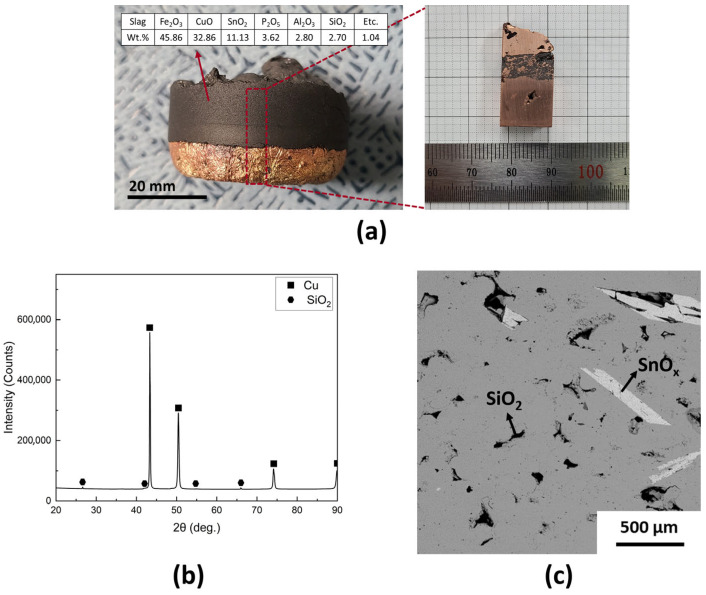
Recovered metal after oxidative refining process without slag former. (**a**) Macrograph of the obtained sample, (**b**) XRD pattern, and (**c**) micrograph of the recovered metal.

The crude metal was subjected to an oxidative refining process, during which a gas mixture of 1 L/min of O_2_ and 1 L/min of N_2_ was injected into the melt at 1300 °C for 10 min. As shown in Figure 3a, the sample obtained after refining exhibited two distinct layers: slag in the upper layer and metal in the lower layer. However, the two layers were not fully separated and remained partially attached. To investigate the composition of the slag, XRF analysis was performed, confirming that a significant amount of Fe was oxidized and transferred to the slag layer. Additionally, elements such as Sn, P, Al, and Si were oxidized and also migrated into the slag. However, a notable quantity of Cu was also oxidized and entered the slag layer. The longitudinal section image of the sample, visible in the right panel of Figure 3a, shows a mixed layer where the metal and slag are not completely separated. XRD analysis of the metal phase revealed strong Cu peaks, along with a low-intensity SiO_2_ phase (Figure 3b). Impurities such as Fe and P were not detected in the metal. The SEM micrograph shown in Figure 3c further confirms the presence of silicon oxides, identified as SiO_2_ based on XRD and EDX results, dispersed throughout the Cu matrix. Additionally, tin oxide was observed to remain within the Cu metal rather than migrating into the slag layer.

This initial oxidative refining process, conducted without the use of slag formers, highlighted the limitations in achieving complete impurity removal and effective separation of slag from metal. While elements such as Fe, P, and Si were successfully oxidized and transferred to the slag, the metal and slag layers remained partially attached, and residual impurities, including Si and Sn oxides, persisted within the Cu phase.

**Figure 4:**
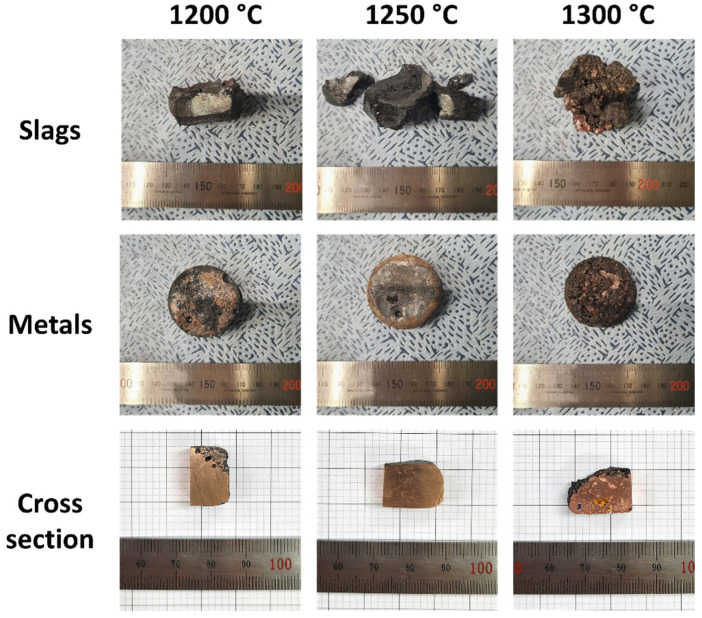
Macrographs of the obtained samples after the oxidative refining process with the FeO–CaO–SiO_2_ slag system.

To enhance slag formation, facilitate impurity removal, and improve metal–slag separation between the slag and metal layers, slag formers such as CaO and SiO_2_ were introduced, and the oxidative refining process was continued. It was anticipated that FeO, formed from the oxidation of Fe within the crude metal, would combine with the added CaO and SiO_2_, resulting in the formation of an FeO–CaO–SiO_2_ slag system. Figure 4 shows the appearance of the samples recovered after oxidative refining at 1200 °C, 1250 °C, and 1300 °C using the 40FeO–30CaO–30SiO_2_ slag system. The first row illustrates the slag, the second row displays the metal alloy, and the last row presents cross-sectional images of the metal alloy. In all three temperature conditions, the slag layer was well-formed, and, unlike the refining process without slag formers, the slag and metal layers were fully separated. At both 1200 °C and 1250 °C, a glassy slag was observed, and the separation between slag and metal was smooth. Notably, after processing at 1250 °C, the surface of the metal sample appeared smooth with no visible slag contamination. In contrast, for the sample processed at 1200 °C, a slight mixing of slag with the metal layer was observed in the cross-sectional view. At 1300 °C, a significant amount of Cu was detected in the slag layer, indicating partial mixing. The surface of the metal sample appeared oxidized, rough, and darker in color. Despite this, the cross-sectional image showed a clear separation between the slag and metal, though pores were observed within the metal phase.

**Figure 5:**
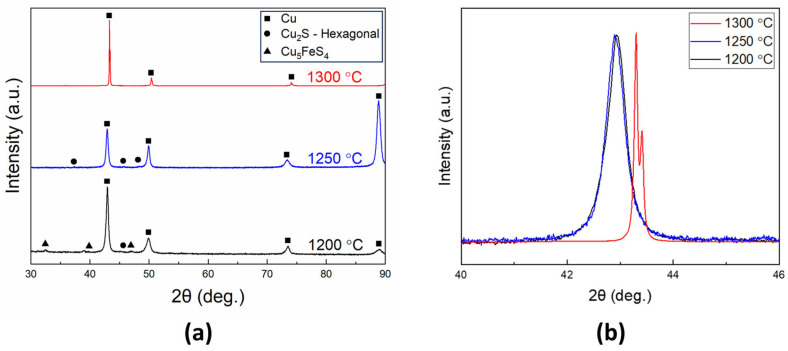
(**a**) XRD patterns and (**b**) normalized Cu (111) peak of the recovered metals after the oxidative refining process at 1200 °C, 1250 °C, and 1300 °C.

To determine the composition of the recovered metals after the oxidative refining process, XRD analysis was conducted. Figure 5 presents the XRD patterns of the metal samples recovered at different processing temperatures. After refining at 1200 °C, peaks corresponding to Cu_5_FeS_4_ and Cu_2_S phases were observed alongside the main Cu peaks. At 1250 °C, the XRD pattern showed dominant Cu peaks along with Cu_2_S phase peaks. However, after processing at 1300 °C, only intense Cu peaks were detected, indicating the removal of secondary phases. Figure 5b presents the normalized Cu (111) peak with an enlarged scale. At both 1200 °C and 1250 °C, the Cu (111) peak was shifted toward a lower 2θ angle compared to the standard Cu (111) position in the powder diffraction pattern, suggesting an expansion in the lattice parameter. Additionally, broadening of the Cu (111) diffraction peak was observed, which is attributed to micro-strain within the crystal structure. In contrast, for the sample processed at 1300 °C, the Cu (111) peak shifted toward a higher 2θ angle, aligning closely with the standard Cu (111) position at 43.3 °. This shift indicates a reduction in the lattice parameter at 1300 °C. Moreover, the peak became sharper, suggesting a reduction in micro-strain within the Cu matrix at this temperature.

**Figure 6:**
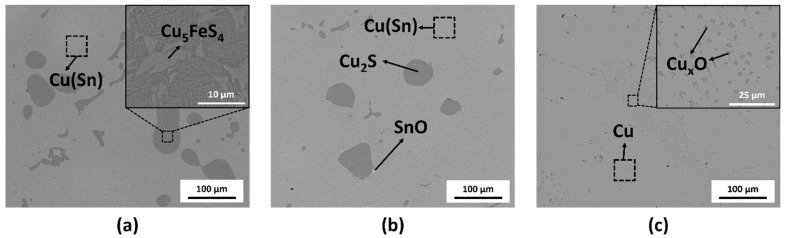
SEM micrographs of the recovered metals after the oxidative refining process at (**a**) 1200 °C, (**b**) 1250 °C and (**c**) 1300 °C.

The microstructure of the recovered metal alloy after oxidative refining was observed using SEM, and the micrographs are presented in Figure 6. As shown in Figure 6a, the metal refined at 1200 °C contained a Cu matrix with Sn, as confirmed by EDX analysis. Within the Cu matrix, dark gray phases were observed, consisting of Cu_5_FeS_4_ and Cu_2_S phases. Figure 6b shows that after processing at 1250 °C, the metal alloy had a Cu matrix with dissolved Sn and dark gray Cu_2_S phases. Small amounts of SnO were observed at the boundaries of the Cu_2_S phases, although the SnO phase was not detectable in the XRD pattern due to its low concentration. In contrast, Figure 6c displays the SEM micrograph of the metal alloy refined at 1300 °C, revealing small dispersed particles within the Cu matrix, which EDX analysis identified as copper oxides. Sn was no longer detected in the Cu matrix, and no phases containing Fe or S were observed.

The SEM results provide an explanation for the shift in the Cu (111) peak observed in Figure 5b for the metals refined at 1200 °C and 1250 °C. The substitution of Sn into the Cu phase accounts for the peak shift and broadening, as Sn has a larger lattice parameter than Cu. The substitution of Sn into the Cu matrix resulted in an expanded lattice parameter and increased micro-strain, which led to the observed shift and broadening of the Cu (111) diffraction peak. After refining at 1300 °C, Sn was removed from the Cu matrix, leading to the Cu (111) peak shifting back to its standard position in the powder diffraction pattern, along with peak sharpening due to the reduction in micro-strain.

The application of the 40FeO–30CaO–30SiO_2_ slag system in the oxidative refining process demonstrated effective slag formation and improved separation between slag and metal. Particularly at 1250 °C, the metal sample exhibited a smooth surface with no visible slag contamination. While impurity levels were significantly reduced across all processing temperatures, small amounts of Sn, Fe, and S remained after refining at 1200 °C. After processing at 1250 °C, Sn and S were still present, although reduced. By contrast, at 1300 °C, all impurities were effectively removed, with only minor amounts of Cu oxide detected.

To quantitatively analyze the impurity concentrations (Fe, Sn, Si) in the refined metal, ICP analysis was performed, while S content was measured using a C/S analyzer. The impurity contents are summarized in Table 2. At 1200 °C, the Fe content was relatively high at 0.63 wt.%, indicating incomplete oxidation and removal of iron at this temperature. However, the Fe concentration significantly decreased to 0.01 wt.% at both 1250 °C and 1300 °C, suggesting that the slag system becomes highly effective at removing Fe impurities at 1250 °C and above. A similar temperature-dependent trend was observed for Sn. At 1200 °C, the Sn content was 5.49 wt.%, decreasing to 4.56 wt.% at 1250 °C. Complete removal of Sn was achieved at 1300 °C, with levels falling below detectable limits (<0.01 wt.%). This indicates that a temperature of 1300 °C is required for complete Sn removal, as lower temperatures result in significant residual Sn content. Si was not detected (<0.01 wt.%) at any of the temperatures tested, confirming that the slag system was highly effective in removing Si impurities under all conditions. However, S removal proved more challenging. At 1200 °C, the S content was 0.61 wt.%, slightly increasing to 0.80 wt.% at 1250 °C. Nevertheless, at 1300 °C, the S content dropped dramatically to 0.01 wt.%, indicating that higher temperatures are critical for effective sulfur removal.

The refining phenomena can be understood in terms of selective oxidation reactions occurring during the process. Fe impurities are readily oxidized according to the following:

$$\;Fe\;\left(l\right)+\;\frac{1}{2}O_{2}\left(g\right)\to FeO\;(s/l),$$

which subsequently combines with CaO and SiO_2_ to form stable FeO–CaO–SiO_2_ slags. Sn impurities are oxidized to SnO_2_ as follows:

$$\;Sn\;\left(l\right)+\;O_{2}\left(g\right)\to SnO_{2}\;\left(s\right),$$

though their transfer efficiency into the slag is lower, explaining the residual Sn observed at 1200–1250 °C. S is removed mainly through the oxidation of sulfides (SO_2_):

$$\;{Cu}_{2}S\;\left(l\right)+\;O_{2}\left(g\right)\to 2Cu\;\left(l\right)+SO_{2}\;\left(g\right),$$

$$FeS\;\left(l\right)+\frac{3}{2}O_{2}\left(g\right)\to FeO\;\left(s\right)+SO_{2}\;\left(g\right).$$

These reactions account for the progressive decrease in S content with increasing temperature. Cu, by contrast, has a lower tendency to oxidize under the given conditions; although Cu_2_O may form, it is partially reduced and remains in the metallic phase, ensuring the recovery of high-purity Cu.

Overall, the results underscore the critical role of temperature in the impurity removal process. While Fe and Si were effectively eliminated at all tested temperatures, the complete removal of Sn and S was only achieved at 1300 °C. The 40FeO–30CaO–30SiO_2_ slag system demonstrated high efficiency at elevated temperatures, with near-total impurity removal at 1300 °C, where most impurities were reduced to undetectable levels. These findings highlight the importance of optimizing temperature in the oxidative refining process to maximize impurity removal and recover high-purity metals from electronic waste.

## 4. Conclusions

This study investigated the effectiveness of the 40FeO–30CaO–30SiO_2_ slag system in the oxidative refining of Cu-based metal alloy recovered from copper-containing sludge. The results demonstrated that temperature plays a pivotal role in impurity removal, significantly influencing the efficiency of the refining process. At 1200 °C, while Fe and Si impurities were effectively removed, substantial amounts of Sn and S remained in the Cu metal alloy. Increasing the temperature to 1250 °C led to further reduction in Sn and S, but complete removal of these impurities was only achieved at 1300 °C. At this temperature, nearly all impurities, including Fe, Sn, and S, were reduced to undetectable levels, and only minor amounts of Cu oxide were observed. The findings also revealed that at 1250 °C, the refined metal displayed a smooth surface with no slag contamination, whereas at 1200 °C, some slag remained mixed with the metal. The highest temperature, 1300 °C, proved most effective, as the metal and slag were completely separated, and impurities were efficiently removed.

In conclusion, the 40FeO–30CaO–30SiO_2_ slag system demonstrated excellent performance in impurity removal, particularly at higher temperatures. The complete elimination of Sn and S at 1300 °C underscores the importance of temperature optimization in the oxidative refining process to achieve high-purity metal recovery from electronic waste. These results contribute valuable insights into refining practices for the sustainable recycling of valuable metals from copper-containing sludge.

## Figures and Tables

**Table 1:** Estimated chemical composition of the crude metal alloy obtained after smelting.

Elements (wt.%)	Fe-Rich Phase	Cu-Rich Phase	Overall Alloy
Cu	18.0	79.2	71.5
Fe	73.4	8.6	16.9
Sn	0.6	9.9	8.7
P	5.2	0.7	1.3
Si	2.0	0.5	0.7
S	0.9	1.0	1.0

**Table 2:** Contents of Fe, Sn, and Si measured by ICP analysis and S content measured by C/S analyzer.

Wt.%	Fe	Sn	Si	S
1200 °C	0.63	5.49	<0.01	0.61
1250 °C	0.01	4.56	<0.01	0.80
1300 °C	0.01	<0.01	<0.01	0.01

## Data Availability

The original contributions presented in this study are included in the article material. Further inquiries can be directed to the corresponding author.
